# Burden and determinants of renal dysfunction and in-hospital mortality among acute stroke patients in Ethiopia: A hospital-based observational study

**DOI:** 10.1097/MD.0000000000039140

**Published:** 2024-07-26

**Authors:** Ermiyas Wondimagegn Nigussie, Eyob Girma Abera, Maekel Belay Woldemariam

**Affiliations:** a Department of Internal Medicine, Jimma University, Jimma, Oromia, Ethiopia; b Department of Public Health, Jimma University, Jimma, Oromia, Ethiopia; c Jimma University Clinical Trial Unit, Jimma, Oromia, Ethiopia.

**Keywords:** acute stroke, cerebrovascular disease, Ethiopia, renal dysfunction, serum creatinine

## Abstract

Stroke, a leading global cause of mortality and neurological impairment, is often complicated by renal failure, exacerbating in-hospital risks and mortality. Limited understanding exists regarding renal failure prevalence in Ethiopian acute stroke patients. This study examines renal function abnormalities in acute stroke patients at Jimma Medical Center (JMC). A hospital-based cross-sectional study was conducted at JMC from December 5, 2023, to March 15, 2024. A structured data collection tool was developed after comprehensive review of pertinent literature, encompassing variables pertinent to the study objectives. Following data quality assurance, information was coded and inputted into EpiData version 3.1, subsequently analyzed using Statistical Package for Social Sciences (SPSS) version 26.0. Multivariable logistic regression analysis was performed to adjust for confounding variables, with statistical significance set at *P* < .05. The mean age of participants was 60.5 ± 15.5 years, with 129 (64.5%) being male. Forty-five participants (22.5%, 95% confidence interval [CI] = 16.9, 28.9) exhibited renal dysfunction. Advanced age (≥70 years), hypertension, diabetes mellitus (DM), cardiac disease, history of transient ischemic attack (TIA)/stroke, and hemorrhagic stroke type were identified as significant predictors of renal dysfunction among hospitalized stroke patients. The mortality rate was 3.7 times higher in stroke patients with renal dysfunction compared to those with normal renal function (adjusted odds ratio [AOR] = 3.7, 95% CI: 1.41, 6.22). Renal function abnormalities were prevalent among hospitalized acute stroke patients, emphasizing the significance of renal dysfunction as a frequent comorbidity. Older age, hypertension, DM, cardiac disease, history of TIA/stroke, and hemorrhagic stroke type emerged as statistically significant predictors of renal dysfunction. Furthermore, renal dysfunction was identified as a significant predictor of in-hospital mortality following stroke.

## 1. Introduction

According to the World Health Organization (WHO), a stroke is defined by rapidly emerging clinical evidence of localized (or global) neurological impairment of brain function, with sudden onset symptoms lasting for more than 24 hours or resulting in death, with no apparent cause other than a vascular origin.^[1]^ Stroke ranks as the second most common cause of mortality globally and is the leading cause of neurological impairment.^[2]^ Approximately 13% of strokes are hemorrhagic, where a blood vessel bursts and blood accumulates in the brain, while the remaining 87% are ischemic, characterized by a reduced blood supply to the brain.^[3]^

Acute stroke patients have a hospital mortality and morbidity rate ranging from 7.6% to 30%, with neurological fatalities accounting for around 80% of these deaths, and non-neurological causes accounting for about 17%.^[1]^ Annually, 15 million people worldwide experience a stroke; of these, 5 million die, 5 million suffer from permanent disability, placing a significant burden on families and communities, and only 5 million achieve optimal recovery.^[4]^

Common risk factors for stroke include advanced age, smoking, diabetes, dyslipidemia, hypertension, and heart disease.^[5]^ Renal failure is also a major risk factor for cardiovascular disorders, including stroke.^[6,7]^ According to the Kidney Early Assessment Program,^[7]^ patients with renal dysfunction face a higher risk of myocardial infarction or stroke. Additionally, there is a graded correlation between renal dysfunction and an increased risk of cardiovascular events, such as stroke.^[8]^ Patients who have had a stroke are more likely to develop kidney disease, which can elevate their risk of another stroke and result in poorer long-term outcomes.^[9]^ Many countries are seeing a rise in high blood pressure, hyperglycemia, dyslipidemia, and obesity, contributing to an increase in both strokes and renal dysfunction.^[10]^

The prevalence of renal dysfunction in ischemic stroke patients ranges from 23.9% to 51.3%,^[11,12]^ significantly higher than the age-dependent prevalence rates in the general population, which are between 8% and 16%.^[13,14]^ Evidence indicates that is an independent risk factor for cardiovascular events, including stroke and myocardial infarction,^[15,16]^ and is associated with worse functional and clinical outcomes in ischemic stroke patients.^[17]^

Acute kidney injury (AKI) has been linked to worsened stroke outcomes and higher mortality rates, with the severity of AKI correlating with increased risk of death.^[18–20]^ Timely management of kidney dysfunction following a stroke could therefore play a crucial role in enhancing post-stroke outcomes and reducing mortality rates. Patients experiencing intracranial hemorrhage coupled with acute renal failure exhibit notably higher in-hospital mortality rates (28.7% vs 22.4%; *P* < .0001) compared to those without acute renal failure.^[21]^ AKI occurring alongside acute stroke is prevalent, with an estimated incidence ranging from 8% to 21%.^[19]^ Notably, stroke patients with severe neurological deficits, cardiac issues like heart failure and atrial fibrillation, ischemic heart disease, hyperglycemia, hypertension, low estimated glomerular filtration rate, or advanced age are particularly susceptible to developing AKI.^[19,22]^ Additionally, renal dysfunction in stroke patients has been associated with factors such as female sex, diabetes mellitus (DM), and physical disability.^[23]^

Stroke has emerged as a prominent cause of hospital admissions and a growing public health concern in Ethiopia.^[24,25]^ Significant delays in both pre-hospital and in-hospital care, along with inadequate management, contribute markedly to the poor prognosis for stroke patients.^[26,27]^ Late presentations and suboptimal care further exacerbate the issue, leading to higher in-hospital mortality rates.^[25,28]^ Despite several studies, no further clinical parameters were measured; reports suggest that renal dysfunction among acute stroke patients in Ethiopia ranges between 18.6% and 35.3%.^[29,30]^ Furthermore, there is limited understanding of the prevalence of renal failure in Ethiopian patients following an acute stroke. Consequently, this current study aims to evaluate the prevalence of renal dysfunction, identify associated factors, and examine in-hospital mortality rates among patients with acute stroke at Jimma Medical Center (JMC).

## 2. Methods

### 2.1. Study design and setting

A hospital-based cross-sectional study was conducted from December 5, 2023, to March 15, 2024, at Jimma Medical Center (JMC) in Jimma Town, located 356 km southwest of Addis Ababa. JMC is one of the largest medical institutions in Ethiopia, serving an estimated population of 20 million in the southwest region. It provides a comprehensive range of medical services, covering almost all major types of healthcare.

### 2.2. Eligibility criteria

All patients with acute stroke admitted to the stroke unit of JMC during the study period who were willing to provide valid consent to participate in the study were included, while patients without serum creatinine levels determined within 24 hours of admission were excluded from the study.

### 2.3. Data collection tool and procedure

A structured data collection tool was designed after reviewing relevant literature to include all the possible variables that address the objectives of the study. Data was collected by trained personnel from the patients (caregivers) and their medical records from admission to discharge or death. Sociodemographic characteristics (age, sex, residence, marital status, religion, educational status, and occupation), family history (hypertension and DM), lifestyle behaviors (smoking, khat chewing, and alcohol drinking habits), clinical features (hypertension, DM, cardiac disease, previous stroke/transient ischemic attack, stroke type, blood pressure, National Institutes of Health Stroke Scale score, and Glasgow Coma Scale score), and laboratory values (random blood sugar, total cholesterol, high-density lipoprotein, low-density lipoprotein, triglycerides, sodium, potassium, chlorine, urine protein, and hemoglobin) were abstracted from patients (caregivers) and medical records.

### 2.4. Operational definition

- Stroke is characterized by rapidly developing clinical signs of localized or global neurological impairment of brain function, with abrupt onset symptoms that last for more than 24 hours or result in death, and no obvious cause other than vascular origin.^[31]^
- Renal dysfunction is defined as having a serum creatinine level >1.2 mg/dL.^[32]^
- Diabetes is a chronic, metabolic disease characterized by elevated levels of blood glucose (or blood sugar), which leads over time to serious damage to the heart, blood vessels, eyes, kidneys and nerves with following criteria:
- A fasting plasma glucose level of 126 mg/dL (7.0 mmol/L) or higher.
- A 2-hour plasma glucose level of 200 mg/dL (11.1 mmol/L) or higher during an oral glucose tolerance test.
- A random plasma glucose level of 200 mg/dL (11.1 mmol/L) or higher in a patient with classic symptoms of hyperglycemia or hyperglycemic crisis.
- Hemoglobin A1c level of 6.5% (48 mmol/mol) or higher.^[33]^
- Hypertension is defined as having a systolic blood pressure (SBP) of 130 mm Hg or higher or a diastolic blood pressure (DBP) of 80 mm Hg or higher, measured on at least 2 separate occasions.
- Prehypertension SBP 120–139 mm Hg or DBP 80–89 mm Hg.
- Mild hypertension (stage 1 hypertension): SBP 140–159 mm Hg or DBP 90–99 mm Hg.
- Severe hypertension (stage 2 hypertension): SBP ≥ 160 mm Hg or DBP ≥ 100 mm Hg.^[34]^
- Obesity is a chronic complex disease defined by excessive fat deposits that can impair health.^[35]^
- Dyslipidemia is defined by abnormal levels of lipids in the blood. Specific criteria include:
- Total cholesterol level of 200 mg/dL (5.2 mmol/L) or higher.
- Low-density lipoprotein cholesterol level of 130 mg/dL (3.4 mmol/L) or higher.
- High-density lipoprotein cholesterol level of <40 mg/dL (1.0 mmol/L) in men or <50 mg/dL (1.3 mmol/L) in women.
- Triglyceride level of 150 mg/dL (1.7 mmol/L) or higher.^[36]^

### 2.5. Data processing and analysis

The principal investigator reviewed the collected data to ensure its completeness and quality before data entry. Coding, recoding, cleaning, and data entry were performed using EpiData V3.1 Software. The data were then exported to and analyzed with the Statistical Package for the Social Sciences (SPSS) version 26 (IBM^®^, New York). Results were presented using tables and figures. For the bivariate analysis, independent variables with a *P* value <.25 were selected as candidates for the multivariable logistic regression analysis. In the multivariable logistic regression, we controlled for confounding variables, considering a *P* value <.05 to be statistically significant.

### 2.6. Ethical consideration

Ethical clearance was obtained from the Ethical Review Board of Jimma University Institute of Health (letter number JUIH/IRB/028/24). Prior to data collection, data collectors provided clear explanations about the study’s aims. Information was collected only after obtaining written informed consent from volunteer patients or their caregivers. Participants were given the right to participate voluntarily and were encouraged to ask questions about the study. To ensure anonymity, participants’ names were not used during data collection, and all personal information was kept entirely confidential. The study adhered to the principles of the Declaration of Helsinki and local regulations regarding the protection of human subjects. All procedures were designed to ensure ethical standards were upheld throughout the study period.

## 3. Result

Two hundred twenty-three patients diagnosed with acute stroke were admitted during the study period. However, the serum creatinine levels of 23 patients were not determined within 24 hours of admission and were therefore excluded from the study. Hence, a total of 200 patients were included in this study.

### 3.1. Sociodemographic characteristics

The mean age of the study participants was 60.5 ± 15.5 years, ranging from 18 to 92 years, with the majority (63%) being 70 years old or older. Most of the patients in this study were male 129 (64.5%). Additionally, 132(66%) participants resided in rural areas (Table 1).

**Table 1:** Sociodemographic characteristics of patients, who were admitted with acute stroke from December 5, 2023 to March 15, 2024, at JUMC, South West, Ethiopia, 2024.

Variables	Categories	Frequency	Percentage
Age (yr)	18–44	33	16.5
	45–65	80	40
	≥66	87	43.5
Sex	Female	71	35.5
	Male	129	64.5
Residence	Urban	68	34
	Rural	132	66
Marital status	Single	13	6.5
	Married	138	69
	Divorced	13	6.5
	Widowed	36	18
Religion	Orthodox	61	30.5
	Protestant	31	15.5
	Muslim	108	54
Education status	Primary	51	25.5
	Secondary	20	10
	Tertiary	64	32
	unable to read/write	65	32.5
Occupation	Civil servant	46	23
	Health worker	3	1.5
	Retired	24	12
	Self-employed	70	35
	Daily labor	8	4
	No work	49	24.5

### 3.2. Prevalence of renal dysfunction and clinical profile

The prevalence of renal dysfunction was 22.5% (95% confidence interval [CI]: 16.9, 28.9%). The minimum and maximum serum creatinine values among the study participants were 0.29 and 3.16 mg/dL, respectively, with a mean ± SD of 1.02 ± 0.49 mg/dL. Among the comorbidities, patients with DM had the highest prevalence of renal dysfunction at 42.9%, followed by cardiac disease (35.3%), and hypertension (30.5%). Although the majority of participants had ischemic stroke (116 or 58%), the prevalence of renal dysfunction was higher among patients with hemorrhagic stroke, at 31% (Table 2).

**Table 2:** Prevalence of renal dysfunction and clinical profile of patients who were admitted with acute stroke from December 5, 2023 to March 15, 2024, to JUMC Southwest, Ethiopia, 2024.

Variables	Categories	Renal function	
		Normal (%) 155 (77.5)	Renal dysfunction (%) 45 (22.5)
Diabetes mellitus	No	127 (84.1)	24 (15.9)
	Yes	28 (57.1)	21 (42.9)
Hypertension	No	66 (91.7)	6 (8.3)
	Yes	89 (69.5)	39 (30.5)
Cardiac disease	No	133 (80.1)	33 (19.9)
	Yes	22 (64.7)	12 (35.3)
Family history	HTN	63 (65.6)	33 (34.4)
	DM	18 (81.8)	4 (18.2)
	Not known	74 (90.2)	8 (9.8)
Stroke type	Ischemic	97 (83.6)	19 (16.4)
	Hemorrhagic	58 (69)	26 (31)
Previous stroke/TIA	No	141 (82.5)	30 (17.5)
	Yes	14 (48.3)	15 (51.7)
NIHSS	Minor	55 (98.2)	1 (1.8)
	Moderate	71 (98.6)	1 (1.4)
	Moderate to severe	18 (51.4)	17 (48.6)
	Severe	11 (29.7)	26 (70.3)
BP	Prehypertension	34 (48.6)	36 (51.4)
	Mild Hypertension	77 (93.9)	5 (6.1)
	Severe Hypertension	44 (91.7)	4 (8.3)
GCS	<15	94 (68.1)	44 (31.9)
	15	61 (98.4)	1 (1.6)

BP = blood pressure, DM = diabetes mellitus, GCS = Glasgow Coma Scale, HTN = hypertension, NIHSS = National Institutes of Health Stroke Scale, TIA = transient ischemic attack.

### 3.3. Behavioral-related factors

Among the 27 patients with a history of cigarette smoking, 10 (37%) had renal dysfunction. Patients who were actively consuming alcohol reported the highest prevalence of renal dysfunction at 47.4%, compared to inactive drinkers (23.5%) and nondrinkers (19.5%; Table 3).

**Table 3:** Behavioral-related characteristics of patients, who were admitted with acute stroke from December 5, 2023 to March 15, 2024, to JUMC Southwest, Ethiopia, 2024.

Variables	Categories	Renal function	
		Normal (%) 155 (77.5)	Renal dysfunction (%) 45 (22.5)
Cigarette smoking	No	155 (77.5)	45 (22.5)
	Yes	17 (63)	10 (37)
Alcohol drinking habits	Non alcoholic	132 (80.5)	32 (19.5)
	Inactive alcoholic	13 (76.5)	4 (23.5)
	Actively alcoholic	10 (52.6)	9 (47.4)
Khat chewing habit	No	86 (84.3)	16 (15.7)
	Yes	69 (70.4)	29 (29.6)

### 3.4. Laboratory test features

Among all patients with renal dysfunction, over half (57.8%) had total cholesterol levels of 200 mg/dL or higher. Additionally, one-fourth of these patients had hyperkalemia, and nearly 7% had hypernatremia at admission. Furthermore, approximately 41(91.1%) of those with renal dysfunction tested positive for protein in their urine (Table 4).

**Table 4:** Laboratory test characteristics at admission for patients admitted with acute stroke from December 5, 2023 to March 15, 2024 at JUMC, Southwest Ethiopia, 2024.

Variables	Categories	Renal function	
		Normal (%) 155 (77.5)	Renal dysfunction (%) 45 (22.5)
RBS (mg/dL)	<200	15 (9.7)	26 (57.8)
	≥200	140 (90.3)	19 (42.2)
Total cholesterol (mg/dL)	<200	147 (94.8)	19 (42.2)
	≥200	8 (5.2)	26 (57.8)
HDL (mg/dL)	<35	26 (16.8)	39 (86.7)
	≥35	129 (83.2)	6 (13.3)
LDL (mg/dL)	<130	142 (91.6)	23 (51.1)
	≥130	13 (8.4)	22 (48.9)
Triglyceride (mg/dL)	<200	153 (99)	40 (88.9)
	≥200	2 (1)	5 (11.1)
Sodium (Na)	<135	37 (23.9)	13 (28.9)
	135–145	117 (75.5)	29 (64.4)
	>145	1 (0.6)	3 (6.7)
Potassium (K)	<3.5	20 (24.4)	1 (2.2)
	3.5–5.4	130 (83.9)	33 (73.3)
	>5.4	5 (3.2)	11 (24.4)
Urine protein (dipstick)	Negative	134 (86.5)	4 (8.9)
	Positive	21 (13.5)	41 (91.1)
HGB	<11	5 (3.2)	4 (8.9)
	≥11	150 (96.8)	41 (91.1)
Chlorine (Cl)	<98	14 (9.0)	1 (2.2)
	≥98	141 (91)	44 (97.8)

HDL = high-density lipoprotein, HGB = hemoglobin, LDL = low-density lipoprotein, mg/dL = milligram per deciliter, RBS = random blood sugar.

### 3.5. Complications of patients after admission

**Figure 1.**
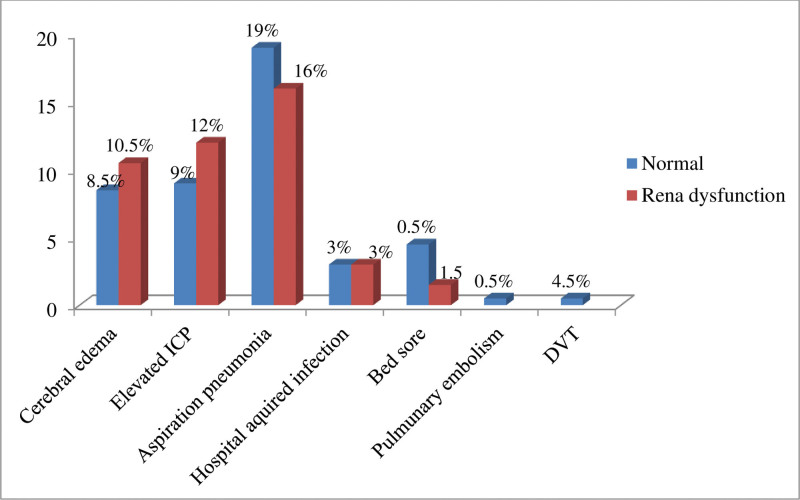
Comparison of complications after admission between normal and those with renal dysfunction. DVT = deep vein thrombosis, ICP = intracranial pressure.

Among admitted patients with acute stroke, the 3 primary complications were aspiration pneumonia 71(35.5%), elevated intracranial pressure (ICP) 42(21%), and cerebral edema 38(19%). When comparing the incidence of complications based on renal function among all admitted cases, patients with renal dysfunction experienced a higher occurrence of cerebral edema (10.5%) and elevated ICP (12%) compared to those with normal renal function. Notably, no cases of deep vein thrombosis or pulmonary embolism were reported among patients with renal dysfunction, despite these complications accounting for 0.5% each among all admitted patients (Fig. 1).

### 3.6. Factors associated with renal dysfunction among acute stroke patients

In the multivariate logistic regression analysis, patient’s age, hypertension, DM, previous stroke/TIA, stroke type, and cardiac disease were associated with renal dysfunction among acute stroke patients. Patients aged 70 years or older had 4.4 times higher odds of renal dysfunction compared to their younger counterparts (adjusted odds ratio, AOR = 4.4, 95% CI: 1.86, 10.2). The odds of renal dysfunction among acute stroke patients with a history of hypertension, DM, and cardiac disease were 3.01, 3.6, and 3.6 times higher, respectively, compared to those without these comorbidities (hypertension: AOR = 3.01, 95% CI: 1.04, 8.68; diabetes mellitus: AOR = 3.6, 95% CI: 1.4, 8.99; cardiac disease: AOR = 3.6, 95% CI: 1.14, 11.63). Additionally, patients with a history of previous TIA/stroke had 3 times higher odds of renal dysfunction than those without (AOR = 3.04, 95% CI: 1.07, 8.6). Regarding the type of stroke, patients with hemorrhagic stroke were 3.8 times more likely to have renal dysfunction compared to those with ischemic stroke (AOR = 3.8, 95% CI: 1.55, 9.48; Table 5).

**Table 5:** Binary and multivariable logistic regression models identifying factors associated with renal abnormalities among patients admitted with acute stroke from December 5, 2023 to March 15, 2024, at JUMC, Southwest Ethiopia, 2024.

Variables	Category	Renal function		COR (95% CI)	*P* value	AOR (95% CI)	*P* value
		Renal dysfunction, 45 (22.5)	Normal renal function, 155 (77.5)				
Age	<70	13 (28.9)	113 (72.9)	1		1	
	≥70	32 (71.1)	42 (27.1)	**6.6 (3.17,13.8**)	**<.001**	**4.4 (1.86,10.2**)	**<.001**
Sex	Female	11 (24.4)	60 (38.7)	1		1	
	Male	34 (75.6)	95 (61.3)	1.9 (0.92,4.14)	.125	1.3 (0.51,3.43)	.325
HTN	No	8 (17.8)	64(41.3)	1		1	
	Yes	37 (82.2)	91 (58.7)	**3.2 (1.42,7.44**)	**.002**	**3.01 (1.04,8.68**)	**.023**
DM	No	22 (48.9)	129 (83.2)	1		1	
	Yes	23 (51.1)	26 (16.8)	**5.2 (2.52,10.6**)	**<.001**	**3.6 (1.4,8.99**)	**<.001**
Cardiac disease	No	31 (68.9)	135 (87.1)	1		1	
	Yes	14 (31.1)	20 (12.9)	**3 (1.4,6.69**)	**.006**	**3.6 (1.14,11.63**)	**.04**
Stroke type	Ischemic	19 (42.2)	97 (62.6)	1		1	
	Hemorrhagic	26 (57.8)	58 (37.4)	**2.3 (1.16,4.49**)	**.03**	**3.8 (1.55,9.48**)	**<.001**
Previous TIA/stroke	No	30 (66.7)	141 (91.0)	1		1	
	Yes	15 (33.3)	14 (9.0)	**5 (2.2,11.5**)	**<.001**	**3.04 (1.07,8.6**)	**.023**
Khat chewing	No	16 (35.6)	86 (55.5)	1		1	
	Yes	29 (64.4)	69 (44.5)	**2.2 (1.13,4.49**)	**.014**	1.6 (0.24,3.44)	.145
Cigarettes smoking	No	35 (77.8)	138 (89)	1		1	
	Yes	10 (22.2)	17 (11.0)	2.3 (0.97,5.50)	.235	0.8 (0.45,2.11)	.33

AOR = adjusted odds ratio, CI = confidence interval, COR = crude odds ratio, DM = diabetes mellitus, HTN = hypertension, TIA = transient ischemic attack.

*P*-value < 0.05.

### 3.7. Factors associated with mortality among acute stroke patients

Out of 200 patients with acute stroke, 34 (17%) died, with the majority (58.8%) having had renal dysfunction. In the multivariate logistic regression analysis, patient’s age, DM, stroke type, cardiac disease and renal dysfunction were associated with in-hospital mortality among acute stroke patients.

The odds of death were significantly higher for acute stroke patients with renal dysfunction (AOR = 3.7, 95% CI: 1.41, 6.22), DM (AOR = 2.9, 95% CI: 1.26, 10.24), cardiac disease (AOR = 4.8, 95% CI: 1.4, 16.5), and those aged 70 or older (AOR = 3.3, 95% CI: 1.25, 9.19) compared to those without these conditions. Additionally, patients with hemorrhagic stroke were 2.9 times more likely to die than those with ischemic stroke (AOR = 2.9, 95% CI: 1.1, 7.98; Table 6).

**Table 6:** Binary and multivariable logistic regression models identifying factors associated with death among patients admitted with acute stroke from December 5, 2023 to March 15, 2024, at JUMC, Southwest Ethiopia, 2024.

Variables	Category	Outcome		COR (95% CI)	*P* value	AOR (95% CI)	*P* value
		Died 34 (17%)	Alive 166 (83%)				
Age	<70	11 (32.4)	115 (69.3)	1		1	
	≥70	23 (67.6)	51 (30.7)	**4.7 (2.14,10.4**)	**<.001**	**3.3 (1.25,9.19**)	**.011**
Sex	Female	8(76.5)	63 (38.0)	1		1	
	Male	26 (76.5)	103 (62.0)	1.4 (0.62,3.11)	.251	1.2 (0.34.2.47)	.454
DM	No	18 (52.9)	133 (80.1)	1		1	
	Yes	16 (47.1)	33 (19.9)	**4 (1.92,9.08**)	**<.001**	**2.9 (1.26,10.24**)	**.031**
Previous TIA/stroke	No	23 (67.6)	148 (89.2)	1		1	
	Yes	11 (32.4)	18 (10.8)	**3 (1.33,7.76**)	**.012**	1.1 (0.31,4.32)	.114
Stroke type	Ischemic	14 (41.2)	102 (61.4)	1		1	
	Hemorrhagic	20 (58.8)	64 (38.6)	**2 (1.07,4.82**)	**<.001**	**2.9 (1.1,7.98**)	**.021**
Cardiac disease	No	23(67.6)	143 (86.1)	1		1	
	Yes	11 (32.4)	23 (13.9)	**2.9 (1.28,6.90**)	**.002**	**4.8 (1.4,16.5**)	**.004**
RBS (mg/dL)	<200	16 (47.1)	25 (15.1)	1		1	
	≥200	18 (52.9)	141 (84.9)	**5 (2.26,11.11**)	**<.001**	0.6 (0.13,2.73)	.332
Total cholesterol (mg/dL)	<200	21 (61.8)	145 (87.3)	1		1	
	≥200	13 (38.2)	21 (12.7)	**4 (1.86,9.79**)	**.004**	0.71 (0.19,3.01)	.454
HDL (mg/dL)	<35	21 (61.8)	44 (26.5)	1		1	
	≥35	13(38.2)	122(73.5)	**4.4 (2.06,9.70**)	**<.001**	3.2 (0.65,15.77)	.252
LDL (mg/dL)	<130	18 (52.9)	147 (88.6)	1		1	
	≥130	16 (47.1)	19 (11.4)	**6.8 (3.01,15.7**)	**<.001**	5.2 (0.81,10.2)	.322
Renal dysfunction	No	14 (41.2)	141 (84.9)	1		1	
	Yes	20 (58.8)	25 (15.1)	**8 (3.60,18.01**)	**<.001**	**3.7 (1.41,6.22**)	**.021**

AOR = adjusted odds ratio, COR = crude odds ratio, DM = diabetes mellitus, HDL = high-density lipoprotein, LDL = low-density lipoprotein, mg/dL = milligram per deciliter, RBS = random blood sugar, TIA = transient ischemic attack.

*P*-value** **<** **0.05.

## 4. Discussion

This study assessed the burden of renal dysfunction and associated factors among 200 acute stroke patients admitted in Ethiopia from December 5, 2023, to March 25, 2024. The prevalence of renal dysfunction among the admitted patients was found to be 22.5% (95% CI: 16.9, 28.9%). This finding is consistent with follow-up studies conducted in Nigeria (24%),^[37]^ Israel (24%),^[38]^ Greece (28.1%),^[18]^ India (24%),^[39]^ and with a INTERSTROKE study (22.9%).^[40]^ However, our finding is lower than studies in Israel (36%).^[41]^ and South-West Nigeria (37%).^[42]^ Similarly, our results are lower than those from a prospective study in Nepal (48.1%)^[20,22]^ and a hospital-based systematic review (32%).^[17]^ These discrepancies may be attributed to differences in study populations, such as varying rates of comorbidities like diabetes and hypertension, and differences in healthcare systems and access, which can affect early detection and management of renal dysfunction. Additionally, variations in diagnostic criteria and methods, environmental and lifestyle factors, genetic predispositions, and socioeconomic conditions between regions could also contribute to the differing prevalence rates.

This study indicated that older age was a significant factor associated with renal dysfunction. Participants who were 70 years of age and older were more likely to develop renal dysfunction compared to those younger than 70 years (AOR = 4.4, 95% CI: 1.88, 10.4). This result aligns with previous studies conducted in Southwest Nigeria,^[42]^ INTERSTROKE study,^[40]^ Greece,^[20]^ and India,^[39]^ which also found that respondents in older age groups were more likely to develop renal dysfunction. The possible explanation for this could be that older stroke patients often have multiple comorbidities and may be frail, increasing their risk of complications such as renal dysfunction.

Renal abnormalities are associated with chronic illness comorbidities in acute stroke patients. Hypertension was the most predominant predictor of renal dysfunction. In this study, 128 (64%) stroke patients had a history of hypertension, and 39 (30.5%) of them developed renal dysfunction. This rate is lower than the findings from Greece (77.6%)^[20]^ and Israel (88.7%).^[38,41]^ This variation may be due to differences in potential risk factors for hypertension, such as smoking, dietary habits, aging, lack of physical activity, and urbanization, which are more prevalent in developed countries compared to developing countries like Ethiopia. Additionally, the proportion of behavioral risk factors (alcohol consumption, smoking status, and khat use) in this study is low. However, after controlling for confounding factors with multivariate analysis, a history of hypertension remained a statistically significant predictor of renal dysfunction among acute stroke patients. Respondents with a history of hypertension were more likely to have renal dysfunction compared to those without hypertension (AOR = 3.01, 95% CI: 1.04, 8.68). This finding aligns with studies conducted in Greece,^[20]^ the INTERSTROKE study of 27 countries,^[40]^ and Ethiopia.^[29]^

Respondents with a history of DM were more likely to have renal dysfunction compared to those without a history of DM. Among the 49 (24.5%) patients with a history of DM, nearly half (47%) developed renal dysfunction. Additionally, this study reported that stroke patients with DM were 3.6 times more likely to develop renal dysfunction than those without DM (AOR = 3.6, 95% CI: 1.4, 8.99). This result is consistent with studies conducted in India^[39]^ and Greece.^[20]^

Similarly, stroke patients with cardiac disease are more prone to developing renal dysfunction. According to this study, of the 34 (17%) patients with cardiac disease, about 41% developed renal dysfunction. Additionally, stroke patients with cardiac disease were 3.6 times more likely to develop renal dysfunction than those without cardiac disease (AOR = 3.6, 95% CI: 1.14, 11.63). This finding is supported by a study in Greece, where 28% of stroke patients with renal dysfunction had cardiac disease,^[20]^ and a systematic review.^[40]^ This implies that cardiac diseases can lead to decreased cardiac output, which reduces blood flow to the kidneys, impairing their ability to filter waste products and maintain fluid and electrolyte balance.^[43]^ Additionally, decreased renal perfusion can cause ischemic injury to the kidneys.

Stroke type is another significant factor associated with renal dysfunction. In this study, 84 (42%) patients had hemorrhagic stroke, and of those, 26 (57.8%) developed renal dysfunction. Additionally, indicated that patients with hemorrhagic stroke were 3.8 times more likely to develop renal dysfunction compared to those with ischemic stroke (AOR = 3.8, 95% CI: 1.55, 9.48). Our findings are comparable to studies in India^[39]^ and Nigeria,^[37]^ which reported a prevalence of renal dysfunction of 36% and 38.7% among patients with hemorrhagic stroke, respectively. This is because of hemorrhagic stroke can lead to hemodynamic instability, causing fluctuations in blood pressure.^[44]^ Acute changes in blood pressure can impact renal perfusion, potentially leading to ischemic injury and renal dysfunction.^[45]^

The sixth variable that was found to be associated significantly with renal dysfunction was a previous history of TIA/stroke. In this study, 29 (14.5%) patients had a previous TIA/stroke history, and among them, 15 (33.3%) developed renal dysfunction. Furthermore, patients with a history of previous TIA/stroke had 3 times higher odds of renal dysfunction compared to those without (AOR = 3.04, 95% CI: 1.07–8.6). Consistently, a study from Japan revealed that patients with a history of previous TIA/stroke were significantly more likely to have renal dysfunction compared to patients without previous TIAs (28.1% vs 15.1%; *P* = .001).^[46]^ This association is likely attributed to the fact that both TIA and stroke are manifestations of cerebrovascular disease, indicating underlying systemic vascular pathology. Vascular damage affects not only cerebral arteries but also renal arteries, potentially leading to impaired renal perfusion and function.^[47]^

In this study, a total of 34 (17%) patients with acute stroke died. The odds of death were significantly higher for acute stroke patients with renal dysfunction (AOR = 3.7, 95% CI: 1.41, 6.22). This result is supported by various studies.^[40,48–50]^ Stroke patients with renal dysfunction were more likely to die in the hospital compared to those without. Factors associated with impaired renal function that may contribute to the adverse outcomes of patients with stroke include insulin resistance, oxidative stress, inflammation, endothelial dysfunction, vascular calcification, and increased plasma levels of fibrinogen and homocysteine.^[51]^

Additionally, our study found that age, history of DM, cardiac disease, and hemorrhagic stroke were significantly associated with acute stroke patient mortality. These findings are supported by studies conducted in Modena,^[52]^ India,^[39]^ Greece,^[53]^ Canada,^[54]^ and France at the Universities of Besancon,^[55]^ which reported that respondents with hemorrhagic stroke and older age were more likely to experience short-term mortality in acute stroke compared to those with ischemic stroke and younger ages. Hemorrhagic stroke can cause a rapid increase in ICP and lead to herniation, which is often fatal.^[56]^

Among the deceased patients, nearly half of them (47%) had DM, which demonstrated a significant association with acute stroke patient mortality (AOR = 2.9, 95% CI: 1.26, 10.24). This finding is supported by various studies.^[57–59]^ High blood sugar levels during a stroke can exacerbate brain damage, leading to increased lactic acid production and contributing to neuronal injury and worse outcomes. Moreover, chronic hyperglycemia in diabetes promotes inflammation and oxidative stress, which can damage blood vessels, increasing their susceptibility to rupture or occlusion and thereby leading to both ischemic and hemorrhagic strokes.^[60,61]^

The last significant predictor of mortality in the model was cardiac disease, with 32.4% of deceased patients having cardiac disease, significantly associated with mortality in acute stroke (AOR = 4.8, 95% CI: 1.4, 16.5). This finding is consistent with results from other studies.^[62–65]^ The increased mortality risk associated with cardiac disease in acute stroke patients may be attributed to reduced cardiac output in patients with heart failure or other cardiac conditions, leading to inadequate perfusion to vital organs, including the brain. This can exacerbate brain injury during a stroke and elevate the risk of mortality. Additionally, cardiac diseases can induce hemodynamic instability, resulting in fluctuations in blood pressure and heart rate. These fluctuations, occurring during a stroke, can further compromise cerebral perfusion, worsen brain injury, and heighten the risk of mortality.^[66,67]^

### 4.1. Strength and limitation

While this study provides valuable insights into factors influencing acute stroke patient mortality, several limitations warrant consideration. Firstly, the study’s small sample size and its reliance on data from a single institution may limit the generalizability of its findings to the broader population. Secondly, the use of varying laboratory standards for measuring renal function across different settings may have introduced bias, potentially underestimating or overestimating the prevalence of renal dysfunction in our study cohort. These limitations underscore the need for larger, multicenter studies with standardized measurement protocols to confirm and further explore the associations identified in this study.

## 5. Conclusion

Our study highlights the significant factors associated with renal dysfunction among acute stroke patients in Ethiopia. Age, along with comorbidities such as hypertension, DM, and cardiac disease, emerged as key predictors of renal dysfunction. Moreover, previous history of TIA/stroke and stroke type was identified as another influential factor. Notably, our findings underscore the heightened mortality risk associated with renal dysfunction in acute stroke patients, emphasizing the critical need for early detection and management of renal impairment in this population. Furthermore, future research endeavors with larger and more diverse cohorts, as well as standardized measurement protocols, are imperative to validate these findings and guide targeted interventions aimed at reducing renal dysfunction-related morbidity and mortality in acute stroke patients.

## Acknowledgments

We would like to acknowledge our study participants and their caregivers who were willing to participate in the study. Their contribution was invaluable. Additionally, we express our appreciation to the dedicated data collectors for their unwavering support, from the initial training sessions to the final submission of the manuscript. Their efforts have been instrumental in the success of this research endeavor.

## Author contributions

**Conceptualization:** Maekel Belay Woldemariam.

**Data curation:** Ermiyas Wondimagegn Nigussie, Eyob Girma Abera.

**Formal analysis:** Eyob Girma Abera.

**Methodology:** Eyob Girma Abera.

**Software:** Eyob Girma Abera.

**Supervision:** Maekel Belay Woldemariam.

**Validation:** Ermiyas Wondimagegn Nigussie, Eyob Girma Abera, Maekel Belay Woldemariam.

**Writing – original draft:** Ermiyas Wondimagegn Nigussie, Eyob Girma Abera.

**Writing – review & editing:** Ermiyas Wondimagegn Nigussie, Eyob Girma Abera, Maekel Belay Woldemariam.
